# Urolithin C alleviates pancreatic β-cell dysfunction in type 1 diabetes by activating Nrf2 signaling

**DOI:** 10.1038/s41387-023-00253-3

**Published:** 2023-12-01

**Authors:** Cheng Luo, Can Hou, Danyi Yang, Tingting Tan, Chen Chao

**Affiliations:** 1 Department of Cardiovascular Surgery, The Second Xiangya Hospital of Central South University, Changsha, 410011 Hunan Province PR China; 2 National Clinical Research Center for Metabolic Diseases, Key Laboratory of Diabetes Immunology, Ministry of Education, and Department of Metabolism and Endocrinology, The Second Xiangya Hospital of Central South University, Changsha, 410011 Hunan Province PR China; 3 Department of Nephrology, The Second Xiangya Hospital of Central South University, Changsha, 410011 Hunan Province PR China; 4 Hunan Key Laboratory of Kidney Disease and Blood Purification, The Second Xiangya Hospital of Central South University, Changsha, 410011 Hunan Province PR China; 5 Department of Immunology, School of Basic Medical Sciences, Central South University, Changsha, 410008 Hunan Province PR China

**Keywords:** Type 1 diabetes, Cell biology

## Abstract

**Aims:**

Type 1 diabetes (T1D) is an autoimmune disorder that destroys insulin-generating pancreatic β-cells. Preserving pancreatic β-cell function is important for treating T1D. Our study aims to explore the mechanism underlying urolithin C (UC)-mediated regulation of β-cell function.

**Methods:**

Non-obese diabetic (NOD) mice were administrated with UC to evaluate UC-mediated protection of T1D. The inflammation of the pancreas islets was examined by hematoxylin and eosin staining. Glucose-stimulated insulin secretion (GSIS) assay and oral glucose tolerance test were applied to evaluate the progression of T1D. MIN6 cells were treated with TNF-α, IL-1β and IFN-γ in the presence of UC. Cell viability was analyzed by CCK-8. Cell apoptosis, proliferation and DNA fragmentation were examined by Annexin V-FITC and PI staining, EdU incorporation and comet assays. Keap1, Nrf2, HO-1 and NQO1 were examined by western blot. Immunofluorescence staining was applied to detect Nrf2 and insulin.

**Results:**

UC administration significantly reduced diabetes incidence, attenuated insulitis, elevated insulin levels and GSIS and reduced blood glucose and AUC in NOD mice. Cytokine treatment suppressed MIN6 cell viability and proliferation but enhanced apoptosis and DNA damage, and these detrimental effects were relieved by UC treatment. Furthermore, UC administration inhibited Keap1 expression and promoted the expression of Nrf2, HO-1 and NQO1 in NOD mice. Nrf2 signaling has been reported to be implicated in preventing the onset of diabetes, and HO-1 and NQO1 are phase II antioxidant enzymes that are regulated by Nrf2 signaling. Cytokine treatment upregulated Keap1 and downregulated Nrf2, HO-1 and NQO1 in MIN6 cells, but it was reversed by UC. The nuclear translocation of Nrf2 was prevented by cytokine treatment, but UC promoted its nuclear translocation. UC-mediated upregulation of Nrf2, HO-1 and NQO1, decreased cell apoptosis and increased proliferation and insulin secretion were abolished by silencing of Nrf2.

**Conclusion:**

UC improves pancreatic β-cell function by activating Nrf2 signaling, thereby alleviating T1D progression.

## Introduction

Type 1 diabetes (T1D) is a chronic autoimmune condition in which the immune system attacks β-cells in the pancreas that causes a failure of producing insulin and subsequent hyperglycemia, which accounts for 5–10% of all diabetes cases [1, 2]. A meta-analysis has shown that the incidence of T1D is 15 per 100,000 people and its prevalence is 9.5% worldwide [3]. Unfortunately, the prevalence of T1D is increasing globally [4]. T1D is characterized by insulitis and destruction of insulin-producing pancreatic β-cells [5]. Taking insulin in combination with proper diet and exercise is the primary treatment for patients with T1D, but there is still no cure for T1D. Investigating the mechanisms underlying T1D development and seeking agents for the prevention of the progression of T1D are crucial for T1D treatment.

Pancreatic β-cells are the primary cells in the islets responsible for synthesizing and secreting insulin to reduce blood glucose level and maintain glucose homeostasis [6]. Autoreactive T cells in the islets mistakenly attack β-cells and cause massive destruction, leading to the onset and development of T1D [7]. Moreover, pancreatic β-cell dysfunction and death facilitate T1D progression [8]. Exosomes derived from T cells deliver microRNAs to β-cells and promote β-cell death, which may accelerate the progression of T1D [9]. Preserving the function of β-cells and preventing its death are beneficial to treat T1D. For instance, targeting the PI3K/Akt signaling to regulate β-cell mass and function prevents diabetes [10, 11].

Urolithins, microflora metabolites obtained from ellagic acid derivatives, exert many biological activities including anti-inflammation [12], antioxidant [13] and anti-tumor activities [14]. Intriguingly, urolithins have shown potential therapeutic effects in diabetes. Urolithin A (UA) enhances insulin sensitivity and glucose tolerance and reduces glucose [15]. In streptozotocin-induced type 1 diabetes, urolithins administration improves cardiac function [16]. Urolithin C (UC) activates glucose-dependent insulin secretion via promoting calcium flux into β-cells, suggesting that UC may improve β-cell function in T2D [17]. However, UC-mediated regulation of β-cell function and its contribution in T1D progression are unknown.

Nuclear factor erythroid 2-related factor 2 (Nrf2) is a transcription factor that regulates the cellular defense against toxic and oxidative insults [18]. Genetic silencing of Kelch-like ECH-associated protein 1 (Keap1), a negative regulator of Nrf2, activates Nrf2 to inhibit T-cell infiltration within or near the islets, ameliorate impairment of insulin secretion and prevent the development of T1D in mice [19], and suppression of the Nrf2 signaling accelerates T1D progression [20]. Maintenance of β cell mass depends on Nrf2 to promote the survival, function, and proliferation of β cells, and Nrf2 activation increases insulin sensitivity and preserves β cell mass. Modulating Nrf2 activity in β cells is a promising therapeutic approach for the treatment of diabetes [18]. Keap1 is identified as the protein responsible for negative regulation of Nrf2, and its overexpression represses the nuclear accumulation and transcriptional activity of Nrf2 [21]. Subsequently, Nrf2 regulates various downstream antioxidation and detoxification enzymes such as NAD(P)H quinone oxidoreductase 1 (NQO1) and heme oxygenase-1 (HO-1) via binding to the antioxidant response element in their promoters [22]. UA represses myocardial fibrosis by activating the Nrf2 signaling [23]; however, the interaction between UC and the Nrf2 signaling in pancreatic β cells is unknown. Thus, the aim of our study is to explore the function and mechanism of UC in the modulation of β-cell function in T1D. We hypothesized that UC might improve β-cell function to slow down T1D progression by regulating the Nrf2 signaling. Our study may shed novel light on β-cell function regulation and provide novel therapeutic agents for T1D.

## Methods

### Non-obese diabetic (NOD) mice

Female NOD mice (*n* = 20 for each group) were purchased from Hunan SJA Laboratory Animal Co., Ltd. For early intervention, mice were administrated with UC (Sigma-Aldrich, Burlington, MA, USA) at 50 mg/kg or vehicle through gavage every day for 4 weeks. For late intervention, mice were administrated with UC or vehicle for 10 weeks. The weight, food intake and blood sugar of mice were weekly measured. Mice were sacrificed after a month, and the pancreas were isolated. Blood was collected, and serum was harvested after centrifugation. Mice were randomly grouped (Random allocation was performed using the table of random number), and experiments involving mice were conducted in a blind manner. The investigator was blinded to the group allocation during experiments. Our study was approved by the Animal Care and Use Committee of the Second Xiangya Hospital of Central South University and conducted in compliance with the Guide for the Care and Use of Laboratory Animals. Gpower was conducted to choose the sample size.

### Diabetes incidence

Diabetes incidence was monitored weekly from 10 to 30 weeks. Levels of blood glucose were measured using Accu-Chek Advantage II Strips (Roche, Basel, Switzerland), and mice with two consecutive glucose levels above 250 mg/dL were considered diabetic.

### Hematoxylin and eosin (H&E) staining, insulitis score and immunohistochemistry (IHC) staining

NOD mice were euthanized, and the pancreas was gently collected from mice in early and late intervention groups. The pancreas was fixed in fresh-prepared 4% paraformaldehyde solution overnight and processed for embedding in paraffin and sectioning at 5 µm. Sections were stained with H&E (Beyotime, Shanghai, China). Insulitis was scored 0–4 following these categories: 0 = clear (no infiltration), 1 = <25% infiltration, 2 = 25~50% infiltration, 3 = 50~75% infiltration and 4 = >75% infiltration. The percentage of each score of insulitis/total pancreas islet in each group was analyzed. For IHC staining, sections were rehydrated, and antigen was retrieved in pH 6.0 antigen retrieval citrate buffer (Abcam, Cambridge, UK). Subsequently, sections were blocked and incubated with an insulin antibody (1:10,000, ab282459, Abcam) overnight and then incubated with a goat anti-rabbit IgG HRP (1:1000, ab6721, Abcam) for 1 h. The signal was visualized by 3,3’-Diaminobenzidine (Solarbio, Beijing, China). The quantitative analysis of insulin expression was performed by using ImageJ software. Relative insulin levels were calculated by the normalization method.

### Cell culture and transfection

Murine pancreatic β-cell line MIN6 was purchased from American Type Culture Collection (ATCC, Rockville, MD, USA) and maintained in DMEM containing 10% FBS (Thermo Fisher Scientific, Waltham, MA, USA). Cells were authenticated by STR profiling and tested for mycoplasma contamination. The siRNA against Nrf2 (si-Nrf2) and scramble siRNA (si-NC) were purchased from GenePharma (Shanghai, China). Cells were treated with a mixture of cytokines (cytomix: 10 ng/mL IFN-γ, 5 ng/mL IL-1β and 5 ng/mL TNF-α. PeproTech, Cranbury, NJ, USA) for 24 h. For UC and verapamil treatment, MIN6 cells were treated with UC at 0, 2.5, 5, 15, 25, 50 or 100 μM or verapamil (Sigma-Aldrich) at 50 µM for 24 h. MIN6 cells were transfected with si-Nrf2 or si-NC via Lipofectamine RNAiMAX (Thermo Fisher Scientific) following the manuals. After 72 h, cells were collected for cytokines and UC treatment.

### Glucose-stimulated insulin secretion (GSIS)

For in vitro GSIS, MIN6 cells were treated with glucose at 2.5 mM (normal glucose) or 20 mM (high glucose) for 1 h. Supernatants were collected and stored at −80 °C. Protein concentration was examined using BCA kit (Beyotime), and total protein was used as a normalization control for insulin. For in vivo GSIS, NOD mice treated with vehicle or UC were administrated with glucose at 2 g/kg through oral gavage. After 30 min, tail blood was collected to measure serum insulin levels. Insulin was measured using Mouse Insulin ELISA (Mercodia, Uppsala, Sweden). Blood glucose was examined using a Medisense glucometer.

### Oral glucose tolerance test (OGTT)

NOD mice treated with vehicle or UC were fasting for 6 h, weighed and administrated with glucose at 2 g/kg through oral gavage. Blood was collected at various time points, and glucose was examined using a Medisense glucometer. The Area Under the Curve (AUC) was calculated.

### Cell counting kit-8 (CCK-8)

MIN6 cells were treated as indicated, and culture medium was removed. CCK-8 (Beyotime) was pre-mixed with fresh culture medium at 1:10, and 100 µL of the mixture was added and incubated for 2 h. The absorbance (450 nm) was examined.

### Cell apoptosis

Annexin V-FITC Apoptosis Detection Kit (CA1020) was provided by Solarbio. Briefly, MIN6 cells were treated as indicated, detached and resuspended in binding buffer. Subsequently, Annexin V- FITC (5 µL) was added and incubated for 5 min protected from light. PI (5 µL) was added, and 400 µL of PBS was added. Cells were analyzed using a flow cytometer immediately.

### Immunofluorescence (IF) staining

MIN6 cells were plated on coverslips and treated with cytokines or cytokines + UC. Cells were fixed in 4% paraformaldehyde and permeabilized in 0.2% Triton X-100 for 15 min. Cells were blocked in 5% normal goat serum and incubated with a Nrf2 antibody (PA5-27882, 1:1000, Thermo Fisher Scientific) overnight. Cells were washed and incubated with a Goat Anti-Rabbit IgG H&L (Alexa Fluor 594, ab150080, Abcam) for 1 h. For IF staining in the pancreas, the pancreas was removed and fixed overnight. Next day, the pancreas was embedded in paraffin and cut into 5-µm sections. Sections were dewaxed and permeabilized in 0.5% Triton X-100 for 30 min. After block, sections were incubated with a Nrf2 antibody (PA5-27882, 1:100, Thermo Fisher Scientific) and an insulin antibody (1:1000, 14-9769-82, Thermo Fisher Scientific) overnight. Subsequently, sections were incubated with a Goat Anti-Rabbit IgG H&L (Alexa Fluor 594, ab150080, Abcam) and a Goat Anti-Mouse IgG H&L (Alexa Fluor 488, ab150113, Abcam) for 1 h. Nuclei were counter-stained with DAPI (Beyotime) followed by mounting and imaging. The quantitative analysis of insulin expression was performed by using ImageJ software.

### Enzyme-linked immunosorbent assay (ELISA)

Insulin secretion was determined using Mouse Insulin ELISA (Mercodia). Briefly, 5 µL of samples were added, and 100 µL of 1× enzyme conjugate solution was added and incubated for 2 h on a shaker. Plates were washed, and 200 µL of TMB was added and incubated for 30 min. Stop solution (5 µL) was added, and the absorbance (450 nm) was measured.

### EdU incorporation assay

MIN6 cells (1 × 10^4^) were plated and treated with cytokines, cytokines + UC or cytokines + verapamil for 24 h. EdU (10 µM) was supplemented into the medium. Cells were fixed in 4% formaldehyde for 15 min. Triton X-100 (0.3%) solution was added to permeabilize cells. The reaction cocktail was prepared, added into cells and incubated for 30 min. DAPI was used to stain DNA, and cells were imaged.

### Comet assay

Comet Assay Kit was provided by Abcam, and DNA damage in MIN6 cells was analyzed using Comet assay following the manual. In brief, MIN6 cells were treated with cytokines, cytokines + UC or cytokines + verapamil for 24 h. Cells were suspended in low-melting agarose and mixed thoroughly at 37 °C. The mixture of cells and agarose was plated on comet slides (Abcam) and gelled. Slides were immersed in pre-chilled lysis solution for 1 h and subjected to electrophoresis. Finally, slides were stained with GelRed Nucleic Acid Gel Stain (Biotium, Fremont, CA, USA).

### Western blot

Islets were isolated from NOD mouse pancreas as previously described [24]. MIN6 cells were treated as indicated, and isolated islets were lysed in lysis buffer, and protein was quantified using BCA kit (Abcam). Nuclear and cytoplasmic fractions were separated using Nuclear/Cytosol Fractionation Kit (BioVision, Milpitas, CA, USA). Subsequently, protein (30 µg per lane) was electrophoresed and transferred. Membranes were blocked and incubated with anti-Keap1 (1:2000, ab227828), anti-Nrf2 (1:500, PA5-27882), anti-HO-1 (1:2000, ab52947), anti-NQO1 (1:1000, ab80588) or anti-β-actin (1:5000, ab8227). After washing, membranes were incubated with the HRP-conjugated secondary antibody (1:10,000, ab6721), and bands were visualized by applying ECL substrate.

### Statistical analysis

All data were analyzed using GraphPad Prism 9 (GraphPad Software, San Diego, CA, USA). Results were shown as mean ± standard deviation (SD). Two-sided Student’s *t*-test between two groups and one-way analysis of variance (ANOVA) followed by Tukey’s post hoc test among multiple groups were conducted to analyze the difference comparison. The correlation of blood glucose or insulin and Keap1, Nrf2, HO-1 and NQO1 was analyzed through the Pearson correlation. The variance is similar between the groups that are being statistically compared. *P* < 0.05 was statistically significant.

## Results

### UC prevented T1D progression in NOD mice

**Fig. 1:**
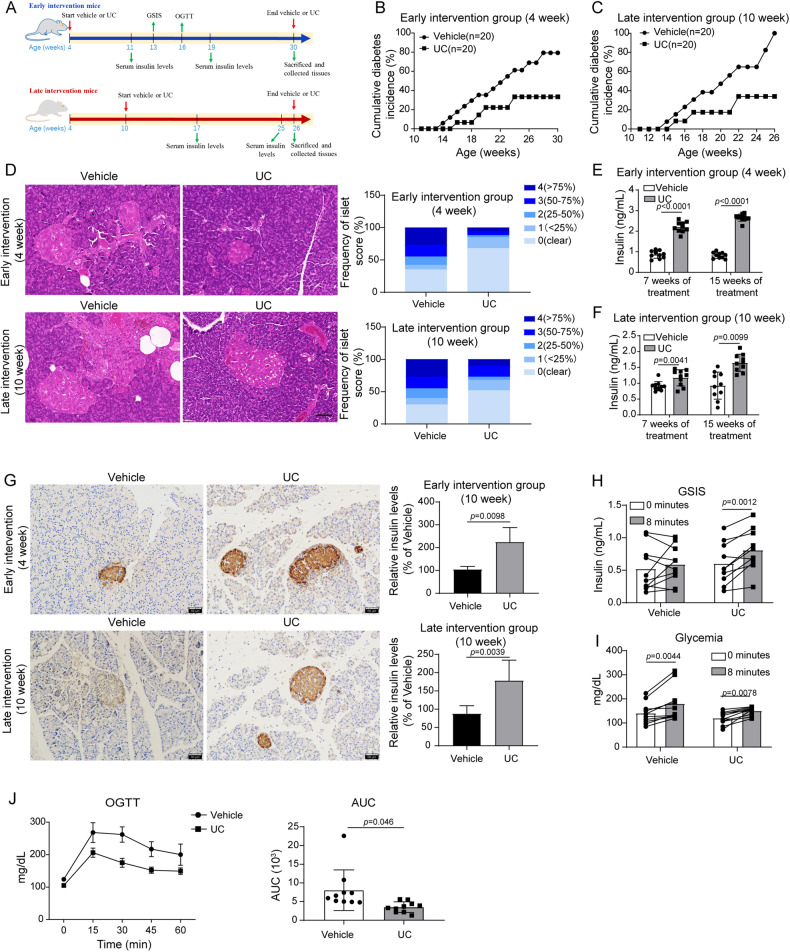
UC prevented T1D progression in NOD mice. **A** A diagram of animal study design. For early intervention, mice were treated with vehicle or UC (50 mg/kg/day) from 4 weeks of age to 30 weeks of age (sacrifice). Serum was collected at 11 and 19 weeks of age, and insulin levels were examined with ELISA. GSIS and OGTT were determined at 13 and 16 weeks of age. For late intervention, mice were treated with vehicle or UC (50 mg/kg/day) from 10 weeks of age to 26 weeks of age (sacrifice). Serum was collected at 17 and 25 weeks of age, and insulin levels were examined with ELISA. **B**, **C** Cumulative diabetes incidence of NOD mice with early and late intervention of UC. Mice treated with vehicle were used as controls (*n* = 20 for each group). **D** H&E staining of the pancreas islets from mice (*n* = 6 for each group. Scale bar: 50 μm). Insulitis was scored 0–4: 0 = clear (no infiltration), 1 = <25% infiltration, 2 = 25~50% infiltration, 3 = 50~75% infiltration and 4 = >75% infiltration. **E**, **F** Serum concentrations of insulin were examined by ELISA (*n* = 10 for each group). **G** IHC staining of insulin in the pancreas islets (*n* = 6 for each group. Scale bar: 50 μm). **H**, **I** Serum insulin and blood glucose levels were evaluated after GSIS treatment (*n* = 10 for each group). **J** Glucose tolerance was evaluated by examining levels of blood glucose at 0, 15, 30, 45 and 60 min and AUC after oral glucose administration (*n* = 10 for each group). Comparison between the two groups was performed by Student’s *t*-test. *p < 0.05, **p < 0.01. All experiments were repeated at least three times.

To evaluate UC-mediated protection of T1D in vivo, UC or vehicle was administrated into NOD mice through gavage. Mice were grouped into early and late intervention groups based on the starting time of UC administration (Fig. 1A). For early intervention, mice were treated with vehicle or UC (50 mg/kg/day) from 4 to 30 weeks of age. Serum was collected at 11 and 19 weeks of age, and insulin levels were examined with ELISA. GSIS and OGTT were determined at 13 and 16 weeks of age. For late intervention, mice were treated with vehicle or UC (50 mg/kg/day) from 10 to 26 weeks of age. Serum was collected at 17 and 25 weeks of age, and insulin levels were examined with ELISA. Mice with two consecutive glucose levels above 250 mg/dL were considered as diabetic disease. Compared to vehicle, UC significantly reduced diabetes incidence in NOD mice, and mice in the early intervention group showed an earlier reduction of diabetes incidence than mice in the late intervention group (Fig. 1B, C). H&E staining showed that UC administration greatly attenuated insulitis in the pancreatic islets, and early intervention of UC exhibited better effects than late intervention (Fig. 1D). Subsequently, we examined insulin levels in mice after 7- and 15-week treatment. We observed that insulin levels in the serum and insulin expression in the pancreatic islets were increasing with UC administration, and late intervention of UC showed moderately enhanced effects compared to early intervention (Fig. 1E–G). Moreover, UC administration enhanced serum GSIS and reduced blood glucose after glucose gavage in NOD mice (Fig. 1H, I). The OGTT test showed decreased blood glucose and AUC in UC-treated mice (Fig. 1J), demonstrating that UC administration enhanced glucose tolerance in NOD mice. Collectively, these observations suggested that UC administration significantly restrained the progression of T1D in NOD mice via elevating insulin level and controlling blood glucose.

### UC protected β-cells from cytokine-induced dysfunction

**Fig. 2:**
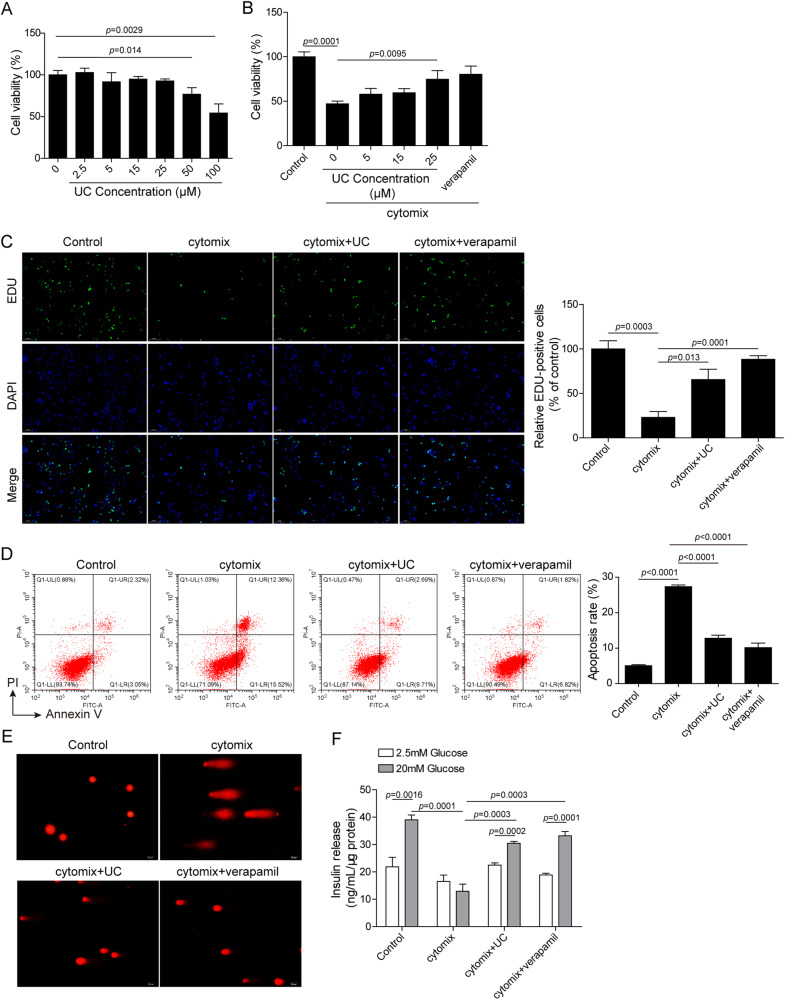
UC protected pancreatic β-cells against cytokine-induced dysfunction. **A** The viability of MIN6 cells treated with UC (0, 2.5, 5, 15, 25, 50 or 100 µM) was analyzed by CCK-8 (*n* = 3). **B** MIN6 cells were treated with cytokines in the presence of UC (0, 5, 15 or 25 μM) or verapamil (50 µM). Cell viability was analyzed by CCK-8 (*n* = 3). In subsequent assays, MIN6 cells were treated with cytokines in the presence of UC (25 μM) or verapamil (50 µM) and divided into four groups: Control, Cytomix, Cytomix + UC and Cytomix + verapamil. **C** Cell proliferation was analyzed by EdU incorporation (*n* = 3). **D** Cell apoptosis was assessed by Annexin V-FITC and PI staining (*n* = 3). **E** DNA fragmentation was assessed by comet assays. (*n* = 3, scale bar: 20 μm). **F** Glucose-induced insulin secretion was examined by ELISA (*n* = 3). Comparison among multiple groups was analyzed by ANOVA (followed by Tukey’s post hoc test). *p < 0.05, **p < 0.01 and ***p < 0.001. All experiments were repeated at least three times.

To investigate whether UC regulates pancreatic β-function in vitro, mouse pancreatic β-cell MIN6 was treated with UC at various concentrations. Low dosages of UC (0, 2.5, 5, 15 and 25 µM) did not affect MIN6 cells viability, whereas 50 and 100 µM of UC showed increasing cytotoxicity (Fig. 2A). Subsequently, MIN6 cells were treated with a cytokine mixture of TNF-α, IL-1β and IFN-γ in the presence or absence of UC, and verapamil, a TXNIP inhibitor for prevention of beta cell loss and diabetes development [25], was used as a positive control in this study. Cytokine treatment markedly inhibited MIN6 cell proliferation, but it was significantly prevented by UC at 15 and 25 µM (Fig. 2B). As 25 µM of UC showed most significant effects (Fig. 2B). Thus, MIN6 cells were treated with UC at 25 µM in subsequent assays. Cytokine treatment reduced EdU incorporation in MIN6 cells, whereas UC and verapamil largely relieved cytokine-mediated suppression of EdU incorporation (Fig. 2C). Moreover, cytokine-induced MIN6 cell apoptosis and DNA fragmentation were also reduced by UC and verapamil (Fig. 2D, E). GSIS assays showed that glucose-mediated insulin secretion was repressed by cytokine treatment, but it was reversed by UC and verapamil (Fig. 2F). These findings suggested that UC improved cytokine-induced pancreatic β-cell dysfunction via facilitating proliferation and insulin secretion and restraining apoptosis and DNA fragmentation.

### UC activated Nrf2 signaling in NOD mice

**Fig. 3:**
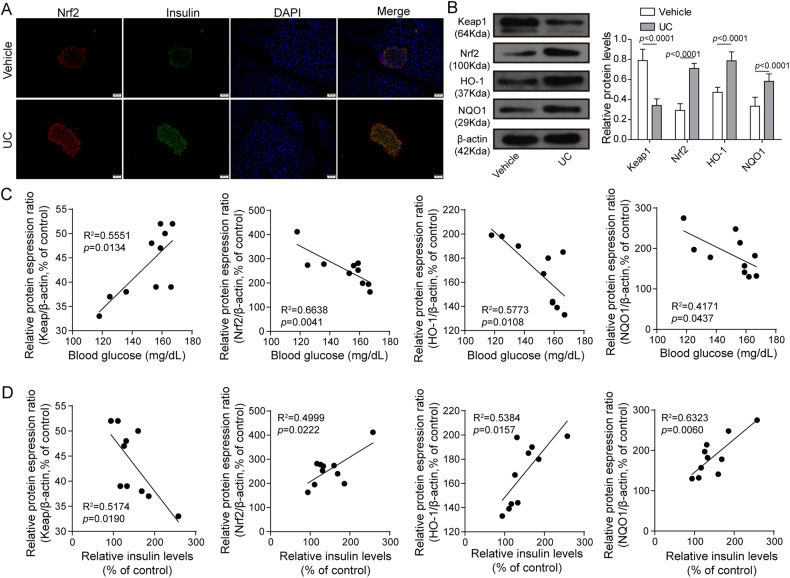
UC activated Nrf2 signaling in NOD mice. NOD mice were administrated with UC (50 mg/kg/day) or vehicle from 4 weeks of age. **A** IF staining of Nrf2 (red) and insulin (green) in the pancreas. Nuclei were stained with DAPI (blue). *n* = 10. Scale bar: 50 μm. **B** Protein levels of KEAP1, Nrf2, HO-1 and NQO1 were determined by western blot. β-actin was used as the loading control. *n* = 10. **C** Pearson correlation analysis of blood glucose levels and the relative expression of Keap1, Nrf2, HO-1 and NQO1. **D** Pearson correlation analysis of serum insulin levels and the relative expression of Keap1, Nrf2, HO-1 and NQO1. Comparison between the two groups was performed by Student’s *t*-test. The correlation of blood glucose or insulin and Keap1, Nrf2, HO-1 and NQO1 was analyzed through the Pearson correlation. *p < 0.05, **p < 0.01. All experiments were repeated at least three times.

As Nrf2 signaling is implicated in preventing onset of diabetes and urolithin A has been reported to activate Nrf2 signaling [26, 27], we examined whether UC regulated Nrf2 signaling in NOD mice. Compared to vehicle, UC dramatically upregulated Nrf2 and insulin in the pancreas (Fig. 3A). We further analyzed the expression of KEAP1, a negative regulator of Nrf2 [28], Nrf2, HO-1 and NQO1, two phase II antioxidant enzymes regulated by Nrf2 signaling [29]. UC evidently downregulated KEAP1 but upregulated Nrf2, HO-1 and NQO1 in the pancreas islets (Fig. 3B). In addition, blood glucose levels were positively correlated with KEAP1 but negatively correlated with Nrf2, HO-1 and NQO1 (Fig. 3C). Conversely, insulin levels were negatively correlated with KEAP1 but positively correlated with Nrf2, HO-1 and NQO1 (Fig. 3D). In conclusion, UC administration activated Nrf2 signaling to control levels of insulin and blood glucose in NOD mice.

### Cytokine treatment-mediated inhibition of Nrf2 signaling was partially relieved by UC

**Fig. 4:**
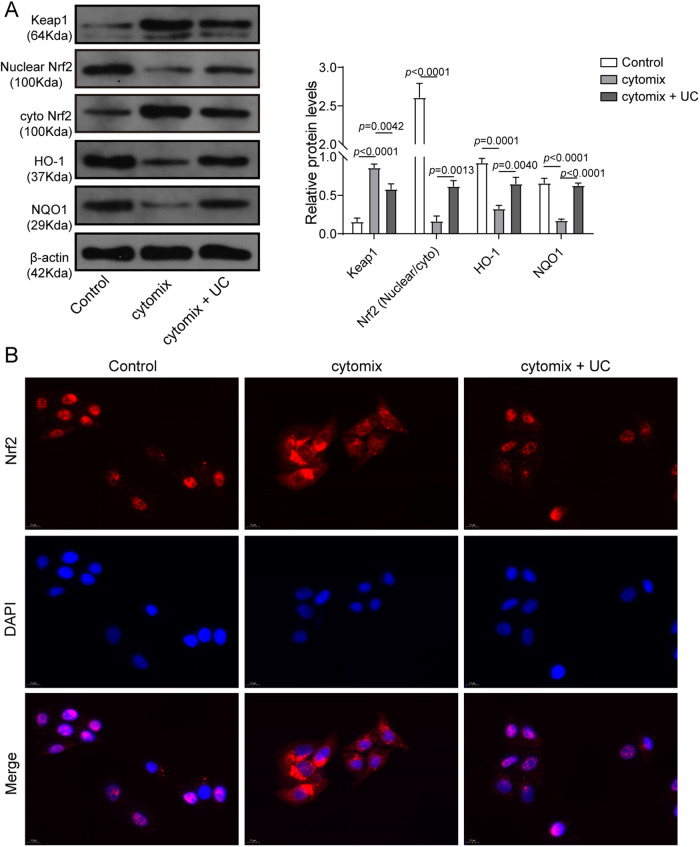
Cytokine treatment-mediated inhibition of Nrf2 signaling was partially relieved by UC. MIN6 cells were treated with cytokines in the presence of UC and divided into three groups: Control, Cytomix and Cytomix + UC. Nuclear and cytosolic fractions were separated. **A** Protein levels of KEAP1, nuclear and cytosolic Nrf2, HO-1 and NQO1 were determined by western blot (*n* = 3). β-actin was used as the loading control. **B** IF staining of Nrf2 (red). Nuclei were stained with DAPI (blue). Scale bar: 10 μm. Comparison among multiple groups was analyzed by ANOVA (followed by Tukey’s post hoc test). *p < 0.05, **p < 0.01. All experiments were repeated at least three times.

We further found that cytokine treatment enhanced KEAP1 expression and downregulated HO-1 and NQO1 in MIN6 cells (Fig. 4A). However, UC treatment partly reversed the expression of KEAP1, HO-1 and NQO1 (Fig. 4A), implying that UC activated Nrf2 signaling in cytokine-treated MIN6 cells. As nuclear translocation is important for triggering Nrf2 signaling, we analyzed Nrf2 abundance in nuclear and cytosolic fractions. It was observed that cytokine treatment promoted Nrf2 abundance in cytosolic fractions but reduced its nuclear abundance, but it was mainly reversed by UC treatment (Fig. 4A). In addition, we found that cytokine treatment restrained nuclear translocation of Nrf2 in MIN6 cells, but simultaneous UC treatment mostly restored its nuclear translocation (Fig. 4B). Therefore, UC treatment activated Nrf2 signaling in cytokine-treated MIN6 cells via regulating the expression of KEAP1, Nrf2, HO-1 and NQO1 and nuclear translocation of Nrf2.

### UC improved cytokine-treated pancreatic β-cell function partly dependent on Nrf2 signaling

**Fig. 5:**
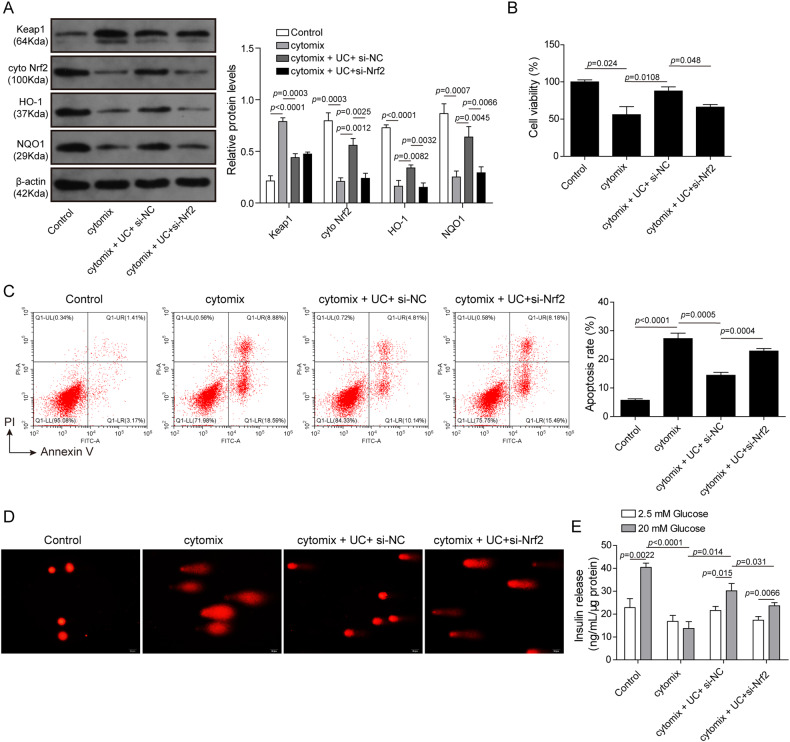
UC improved cytokine-treated pancreatic β-cell function partly dependent on Nrf2 signaling. Nrf2 was knocked down in MIN6 cells, and cells were treated with cytokines in the presence of UC and divided into four groups: Control, Cytomix, Cytomix + UC + si-NC and Cytomix + UC + si-Nrf2. **A** Protein levels of KEAP1, Nrf2, HO-1 and NQO1 were determined by western blot (*n* = 3). β-actin was used as the loading control. **B** Cell proliferation was evaluated by CCK-8 (*n* = 3). **C** Cell apoptosis was assessed by Annexin V-FITC and PI staining (*n* = 3). **D** DNA fragmentation was assessed by comet assays (*n* = 3, scale bar: 20 μm). **E** Glucose-induced insulin secretion was examined by ELISA (*n* = 3). Comparison among multiple groups was analyzed by ANOVA (followed by Tukey’s post hoc test). *p < 0.05, **p < 0.01 and ***p < 0.001. All experiments were repeated at least three times.

To evaluate whether UC-mediated regulation of pancreatic β-cell function is dependent on Nrf2 signaling, Nrf2 was knocked down in MIN6 cells through si-Nrf2 transfection. UC-mediated upregulation of Nrf2, HO-1 and NQO1 in cytokine-treated MIN6 cells was chiefly abrogated by knockdown of Nrf2 (Fig. 5A). However, knockdown of Nrf2 did not affect UC-mediated regulation of KEAP1 expression (Fig. 5A). Moreover, silencing of Nrf2 partly abolished UC-mediated proliferation-promoting and anti-apoptosis effects in cytokine-treated cells (Fig. 5B, C). In addition, silencing of Nrf2 partially counteracted UC-induced DNA fragmentation (Fig. 5D). Furthermore, UC promoted insulin secretion, but it was largely counteracted by Nrf2 knockdown (Fig. 5E). Our findings indicated that UC activated Nrf2 signaling, at least mostly, to improve pancreatic β-cell function.

## Discussion

Diabetes is a serious health condition across the world. If it is not properly controlled, diabetes may cause severe complications such as retinopathy, nephropathy, cardiovascular diseases and foot problems [30]. Although T1D only accounts for 5–10% of diabetes cases, there were over 9 million patients living with T1D in 2017 [31], causing huge global health burden. T1D is a result of the immune system-mediated destruction of pancreatic β-cells, and maintaining its function is key to restraining T1D progression. Here, we uncovered the mechanism underlying the regulation of β-cell function in T1D. Specifically, we identified UC as a regulator to improve β-cell function partially through the KEAP1/Nrf2 pathway, thus repressing the progression of T1D.

UC has been reported to be implicated in activating insulin secretion. Bayle and colleagues identified UC as a glucose-dependent activator of insulin secretion through upregulation of calcium flux into pancreatic β-cells [17]. A further study demonstrated that UC enhanced insulin secretion of β-cells via enhancing glucose-induced activation of the ERK signaling, a key intracellular signaling [32]. These two studies suggest the potential effects of UC on restraining T1D progression via increasing insulin secretion of β-cells but do not provide any direct in vivo evidence. As no other studies regarding the function of UC in T1D have been reported up to now, the mechanisms underlying UC-mediated regulation of β-cell function and T1D progression remain largely unclear. We verified that UC functioned as an activator of insulin secretion. Importantly, we provided direct evidence to demonstrate that in vivo administration of UC in NOD mice could significantly reduce the incidence of T1D and blood glucose levels and slow down its progression, indicating a potential clinical significance of UC as a novel agent for treating T1D.

As pancreatic β-cells are insulin-producing cells, their dysfunction and loss cause insulin deficiency [33]. Proper regulation of β-cell apoptosis and proliferation is essential to maintain its mass and function [34, 35]. UA protects pancreatic β cells from apoptosis via activating autophagy [36]. UC is a glucose-dependent activator of insulin secretion via promoting L-type Ca2+ channel open and Ca2+ influx into pancreatic β-cells [17]. Yin et al. found that UC induced apoptosis and inhibited proliferation of PC12 cells via the mitochondria-mediated pathway [37], suggesting the possibility that UC might regulate β-cell proliferation and apoptosis. Conversely, we found that UC significantly promoted cytokine-treated β-cell proliferation but reduced apoptosis. Furthermore, UC treatment enhanced glucose-mediated insulin generation of cytokine-treated β-cells. Collectively, we first demonstrated that UC suppressed β-cell apoptosis and promoted its proliferation and insulin secretion. The opposing effects of UC on cell apoptosis may result from various cellular microenvironment and downstream signaling pathways, which needs further investigation. Verapamil is known as an inhibitor of voltage-gated L-type Ca2+ channel, and UC is also reported to inhibit the channel, possibly by direct binding. Thus, Nrf2 induction may not the sole mechanism of UC, and its action on the Ca^2+^ channel may be involved. We will focus it in our future studies.

Nrf2 serves key roles in cellular responses to oxidative stress [38]. KEAP1, an endogenous inhibitor of Nrf2, represses Nrf2 activity via promoting its degradation [39]. The KEAP1/Nrf2 signaling plays critical roles in protective responses against inflammatory and oxidative stresses [18, 40]. The activation of the Nrf2 signaling inhibits nitric oxide-induced β-cell apoptosis and significantly enhances insulin secretion in diabetic mice [41]. Nrf2 maintains β-cell mass via inhibiting β-cell death and promoting its proliferation. Besides, Nrf2 promotes β-cell function and proliferation via regulating β-cell mitochondrial biogenesis [42, 43]. Furthermore, Yagishita et al. found that activation of the KEAP1/Nrf2 signaling repressed T-cell infiltration into the islets, ameliorated T1D progression and prevented the onset of T1D in NOD mice [19]. Based on these previous studies, we determined to examine whether UC regulated β-cell proliferation, apoptosis and function dependent on targeting the KEAP1/Nrf2 signaling, at least mostly. Intriguingly, we found that UC activated the Nrf2 signaling by upregulating Nrf2, HO-1 and NQO1 and downregulating KEAP1 to maintain levels of glucose and insulin in vivo. UC regulated proliferation, apoptosis and insulin secretion of cytokine-treated β-cells through the Nrf2 signaling. Thus, the Nrf2 signaling may be activated to intervene T1D. KEAP1 has been reported to regulate the expression of cytokine genes [44], but cytokine-mediated regulation of KEAP1 expression is unknown. Here, for the first time, we found that cytokine treatment could induce KEAP1 expression, but the molecular mechanisms need further investigation.

## Conclusions

In summary, we first demonstrated that UC alleviated pancreatic β-cell dysfunction in T1D, partially dependent on activating Nrf2 signaling to maintain glucose homeostasis, thus preventing the onset of T1D and slowing down its progression. Our findings deepen the understanding of the regulation of pancreatic β-cells in T1D and identify novel agents and targets for T1D treatment. However, the evidence is limited and the translation of these findings to humans is unclear. There are many confounders, such as baseline urolithin levels, other triggers of autoimmunity, age, gender, dietary habit, etc. We consider adopting these steps to promote the translation of our findings to humans. First, the detailed mechanisms by which UC activates the Nrf2 signaling need to be further investigated. Second, the roles of UC and the mechanisms should be explored in human cells. Third, clinical samples should be involved in further studies.

## Data Availability

All data generated or analyzed during this study are included in this published article.
